# Supplementary figures and images for: Heterologous Booster with BNT162b2 Induced High Specific Antibody Levels in CoronaVac Vaccinees
Source: Vaccines (Basel). 2023 Jun 30;11(7):1183. doi: 10.3390/vaccines11071183 (PMC10383528; doi:10.3390/vaccines11071183)

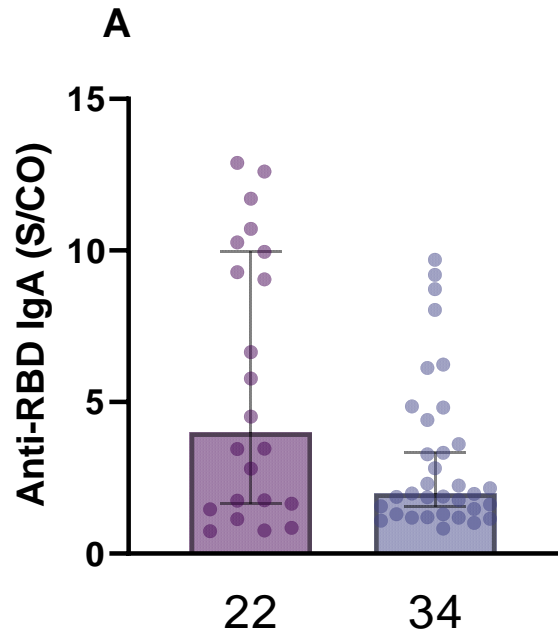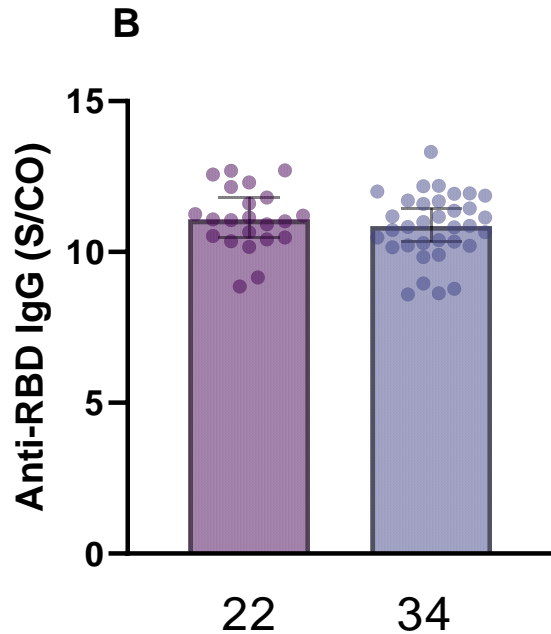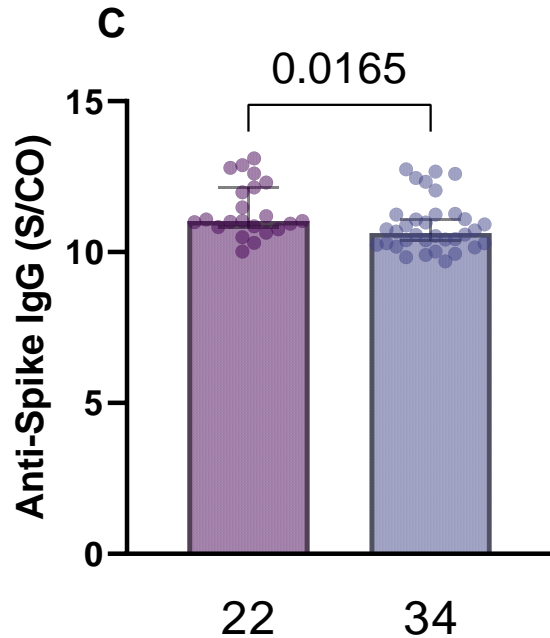

Supplement: Supplementary file 1 [file vaccines-11-01183-s001.zip › Figure S1 - Assessment of the gender influence on anti-RBD IgA and IgG and anti-spike IgG serum levels post third dose.pdf]

## Total vaccines

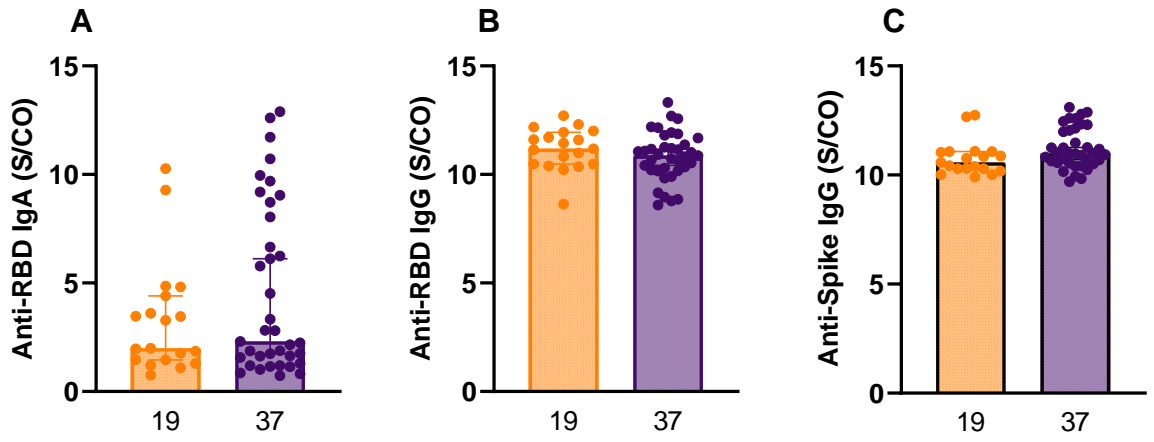

## ChAdOx1 nCoV-19

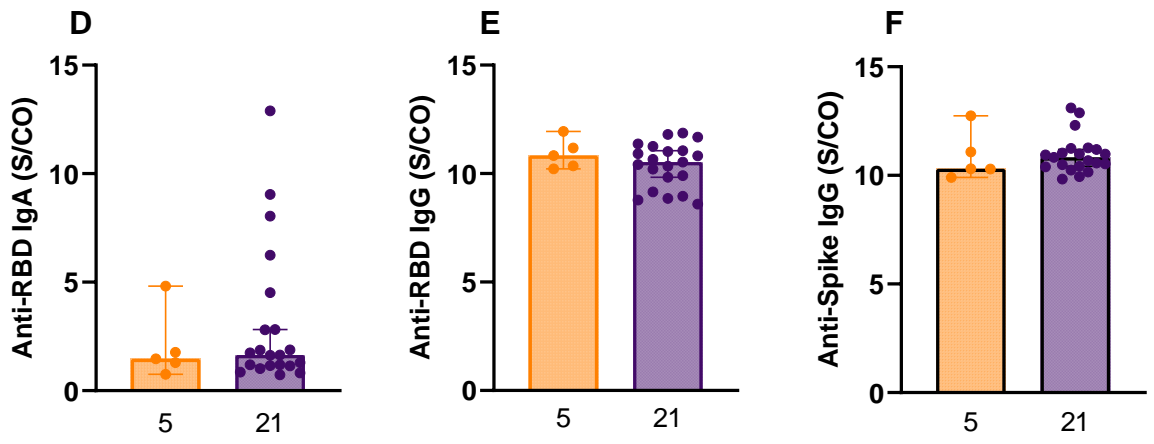

## BNT162b2

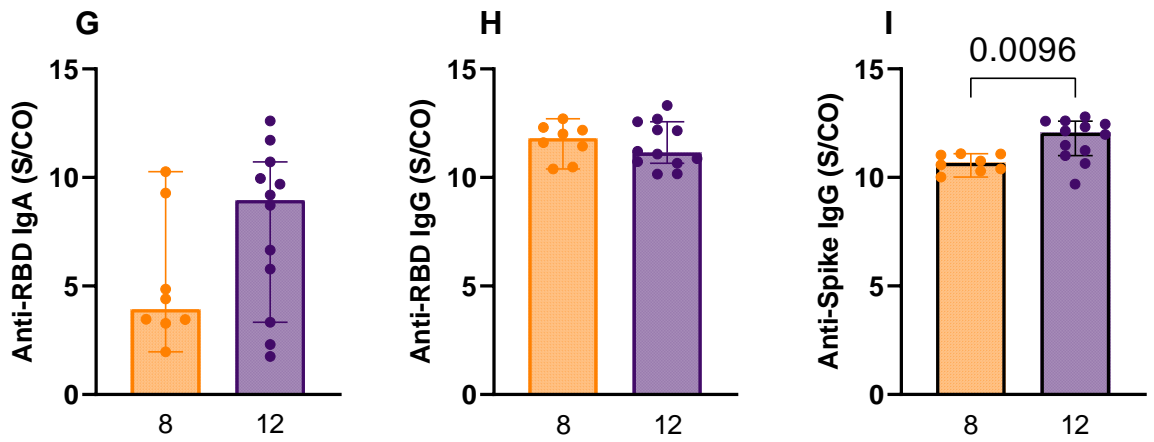

## CoronaVac

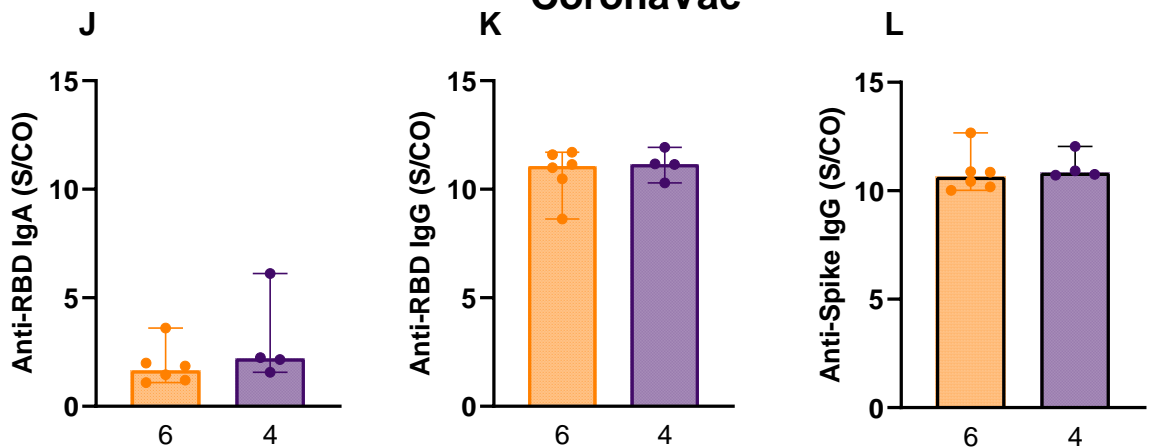

Supplement: Supplementary file 1 [file vaccines-11-01183-s001.zip › Figure S2 - Variation in anti-RBD IgA and IgG and anti-spike IgG serum levels due to comorbidities.pdf]

## Pre-vaccine

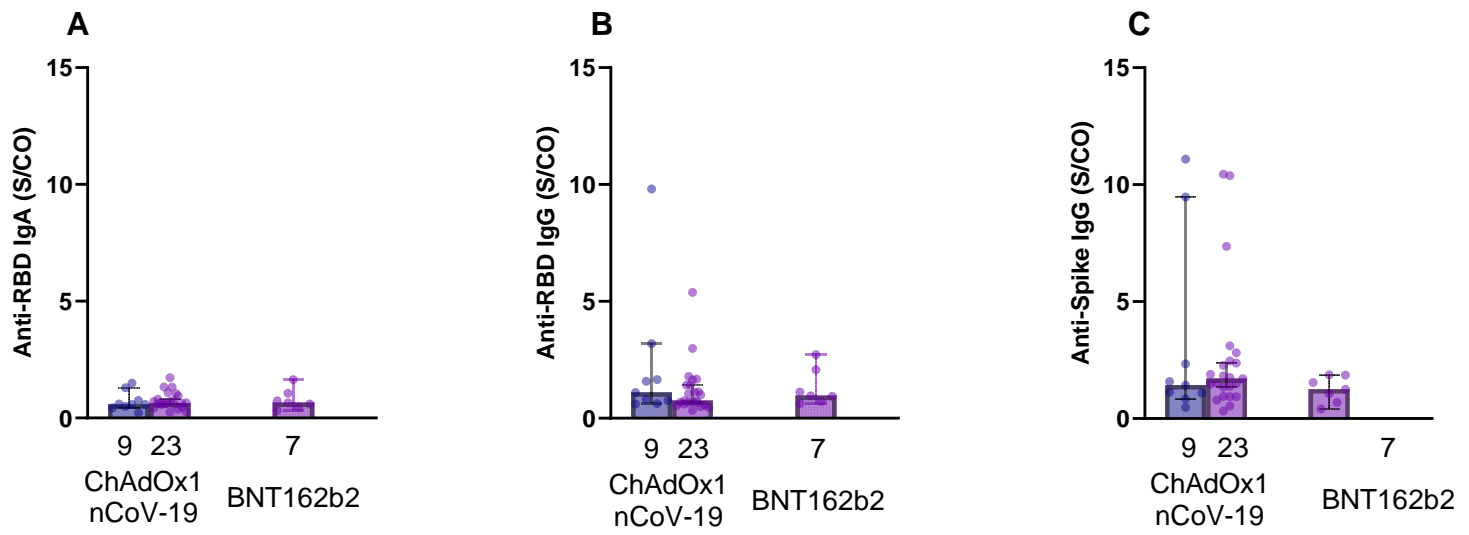

## 1 month post second dose

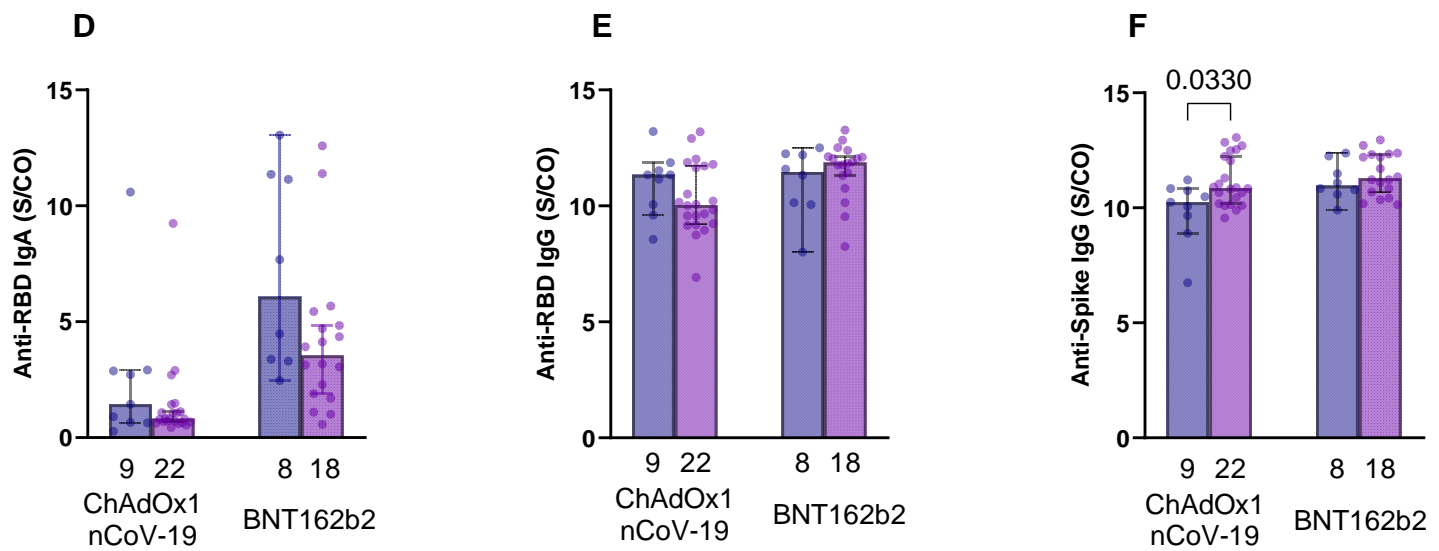

## 1 month post third dose

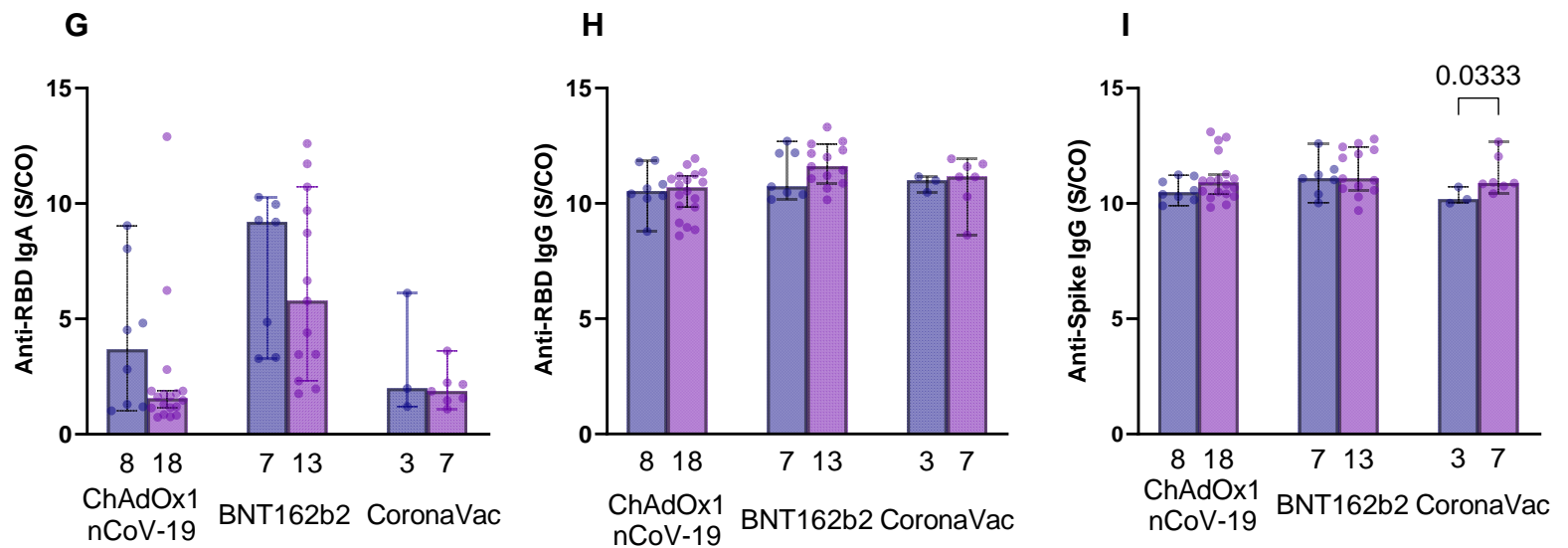

Supplement: Supplementary file 1 [file vaccines-11-01183-s001.zip › Figure S3 - Analysis of anti-RBD IgA and IgG and anti-spike IgG serum production in relation to the history of SARS-CoV-2 infection.pdf]
